# Utilizing vaginal natural orifice to facilitate bowel manipulation during totally intracorporeal ileal conduit construction: a retrospective cohort study

**DOI:** 10.1080/07853890.2025.2453827

**Published:** 2025-01-18

**Authors:** Kaipeng Jia, Shiwang Huang, Zhun Wang, Yuda Lin, Yiduo Bai, Chong Shen, Zhe Zhang, Zhouliang Wu, Yunkai Qie, Hailong Hu

**Affiliations:** a Department of Urology, The Second Hospital of Tianjin Medical University, Tianjin, China; b Tianjin Key Laboratory of Urology, Tianjin Institute of Urology, The Second Hospital of Tianjin Medical University, Tianjin, China

**Keywords:** Female, intracorporeal urinary diversion (ICUD), natural orifice specimen extraction surgery (NOSES), robotic-assisted radical cystectomy (RARC), vagina, surgical technique

## Abstract

**Objective:**

To explore the feasibility and efficacy of the modified technique of totally intracorporeal ileal conduit (IC) construction *via* vaginal approach following robot-assisted radical cystectomy (RARC) in females.

**Methods:**

By comparing the perioperative outcomes of the modified technique with extracorporeal urinary diversion (ECUD), 31 females treated for bladder cancer with RARC and IC from May 2020 to December 2023 were retrospectively analyzed and divided into two groups: the ECUD group (10 patients) and the modified intracorporeal urinary diversion (MICUD group) (21 patients). The modified technique involved performing transvaginal natural orifice specimen extraction surgery (TV-NOSES) after RARC; followed by the transvaginal placement of an Endo-GIA stapler to manipulate the bowel for intracorporeal IC construction.

**Results:**

Both groups’ surgeries were successfully completed by the same surgeon and team. Patients in the MICUD group had shorter total operative time, lower postoperative pain scores, quicker recovery, and shorter hospital stays. The learning curve of the MICUD showed two phases: a learning phase (cases 1-7) and a proficiency phase (cases 8-21). The incidence of postoperative complications between the two groups was similar. The mean follow-up times were 29.3 months (ECUD group) and 22.6 months (MICUD group). In the MICUD group, there was one case of local tumor recurrence, two cases of distant metastasis, including one death from progression; in the ECUD group, one patient had distant metastasis and died from progression.

**Conclusion:**

RARC with MICUD represents a safe, feasible and easy-to-learn minimally invasive surgical approach. Patients experience less trauma and faster recoveries.

## Introduction

Radical cystectomy (RC) is the primary treatment for muscle-invasive bladder cancer (MIBC), very high-risk, and some high-risk non-muscle-invasive bladder cancers (NMIBC) [1]. Robot-assisted radical cystectomy (RARC) has been developed with fewer complications than open surgery and can achieve the same tumor control results [2]. Urinary diversion, as an integral part of radical cystectomy, has also evolved from an extracorporeal urinary diversion (ECUD) to intracorporeal urinary diversion (ICUD) [3,4]. The iROC study reveals that compared to open RC, RARC with ICUD leads to an increase in the days alive and out of the hospital over 90 days [5]. To date, RARC with ICUD is emerging as a direction of RC [2,3]. And ileal conduit (IC) is the most widely utilized urinary diversion technique [6].

According to previous reports, ICUD is mostly performed through a trocar above the pubic symphysis or in the left lower abdomen, through which an Endo-GIA stapler is placed [7,8]. However, compared to ECUD, traditional ICUD may increase surgical complexity and prolong operative time, which is why many surgeons prefer ECUD [3,9,10].

Although the incidence of bladder cancer in men is approximately four times higher than in women, female patients are more inclined to undergo RC [11]. The unique anatomical structures in females may give rise to distinct characteristics in this surgical procedure. And we have modified the ICUD procedure by utilizing the Transvaginal natural orifice specimen extraction surgery (TV-NOSES), followed by the transvaginal placement of an Endo-GIA stapler to manipulate the bowel and perform ileal conduit. We suggest the modified technique may make ICUD more popular. This article aims to investigate the feasibility and efficacy of the modified ICUD (MICUD) construction in this patient population through comparison with ECUD.

## Materials and methods

### Patients and general information

The written informed consents have been obtained from all individual participants for this retrospective study. Retrospective analysis was conducted on the clinical data of 31 female patients who underwent RARC with ileal conduit for bladder cancer at the Second Hospital of Tianjin Medical University from May 2020 to December 2023. Among these patients, 10 underwent ECUD (ECUD group), and 21 underwent MICUD (MICUD group). In this study, all surgeries were performed by the same console surgeon with standardized perioperative patient management. Baseline characteristics and demographic data of the patients were collected, along with perioperative information, to explore the feasibility and efficacy of the modified technique. Tumor characteristics and postoperative follow-up data were also collected to describe short-term tumor outcomes.

### Surgical technique

**Figure 1.**
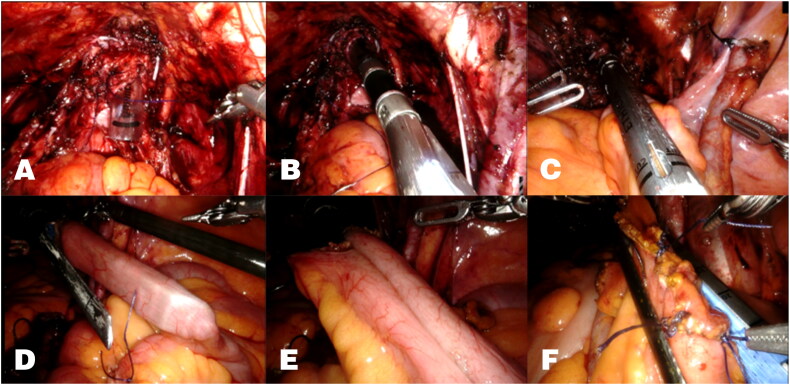
Transvaginal intestinal reconstruction (A) A 12 mm trocar was placed through vagina. (B) Applications of the Endo-GIA stapler was performed through vagina. (C) A segment of ileum was isolated to form the ileal loop. (D and E) Creating a side-to-side anastomosis of the ileum. (F) closure of enterotomy.

For MICUD, before the surgery, the vagina was cleansed daily with a 0.3% iodine solution for a consecutive period of 2 to 3 days. During the surgical procedure, the vagina was packed with iodine-soaked gauze. The patient was placed in the Trendelenburg lithotomy position with an angle of 20–25° after general anesthesia and tracheal intubation. The patient-side cart was positioned on the patient’s side. The port placement was described in Supplementary material. The da Vinci Xi surgical system was utilized to perform RARC with bilateral pelvic lymph node dissection. Subsequently, an intracorporeal IC procedure was performed. The assistant removed the gauze that was packed in the vagina and a EndoCatch was then inserted through the vagina to retrieve all specimens. The surgeon employed a 2-0 V-Loc suture for continuous suturing of the urethral meatus and part of the vagina. After that, the assistant positioned a 12 mm trocar in the vagina (Figure 1A) and inserted an Endo-GIA stapler (Figure 1B) through it. During this procedure, the surgeon can effectively tighten the junction and reduce the gaps between the trocar and the vaginal stump to prevent air leakage. An ileal segment, approximately 15 cm long, was harvested about 15 cm away from the ileocecal valve for the creation of the ileal conduit (Figure 1C). Then, the stapler with a 60-mm vascular load was applied to form a side-to-side anastomosis. Based on our experience, a single staple firing is usually sufficient. If necessary, an additional staple load can be used to expand the lumen (Figure 1D-E). Finally, the remaining bowel opening was closed with the same type of Endo-GIA stapler, ensuring bowel continuity (Figure 1F). Ureteroileal anastomoses were performed using the Wallace method, and the stents were placed into the ureters on each side. If the decision was made to preserve the uterus, the anterior wall of the vagina was incised approximately 3-5 cm for specimen retrieval. After the laparoscopic surgery part, the robotic arm port 3 was enlarged to complete the creation of the stoma.

For ECUD, following the RARC and dissection of the pelvic lymph nodes, the robot was undocked and the specimen was extracted through extension of the camera port *via* a peri or subumbilical 5-10 cm midline incision. The ileal loop was isolated on its mesentery, the bowel was reconstructed with staplers, and the peritoneal defect was closed. Ureteroileal anastomosis were performed by the Bricker technique. The stoma was fashioned in the standard way.

In this study, most patients had both their ovaries and uterus removed along with the bladder. Regarding the removal of the anterior vaginal wall, according to the NCCN guidelines and previous literatures, we routinely do not remove it. It is only resected in cases where there is evidence of tumor involvement in the bladder neck, urethra, or infiltration of the anterior vaginal wall or parametria [12–14]. When the anterior vaginal wall was resected, we used the clamshell closure technique or Mercedes-Benz closure for vaginal reconstruction [15].

### Observation indexes and follow-up methods

Perioperative data were meticulously recorded, while postoperative pain levels were assessed on a 0-10 numerical scale. Routine postoperative assessments included abdominal CT scan and Lower Extremity Venous Ultrasound on the third day after surgery. Postoperative complications within 30 days were graded using the Clavien-Dindo classification and the Comprehensive Complication Index (CCI) [16]. The healing progress of the surgical incision was evaluated using the Vancouver Scar Scale (VSS). Subsequently, patients were vigilantly monitored, with follow-up visits scheduled every 3 months for the first year, every 6 months for the second year, and annually thereafter.

### Learning curve

The learning curves were performed to evaluate operative times of the MICUD group as the cases progressed chronologically. The operative times include total operative time, surgeon console time (SCT), bowel manipulation time and IC creation time. The time required for bowel manipulation includes: establishment of TROCAR through vagina, selection of the intestinal tube, preparation of the ileal segment, restoration of intestinal continuity, and closure of the peritoneal defect. The construction time of IC includes bowel manipulation and uretero-intestinal anastomosis. The learning curve was plotted using cumulative sum (CUSUM) analyses [17]. This approach was utilized to analyze operative times as cases progressed. The CUSUM method calculates the cumulative deviation between each consecutive data point and the mean operative time, enabling the detection of subtle trends that might not be evident with standard analytical methods. The data was represented on a line graph, resulting in a curve.

### Statistical methods

The collected medical records were analyzed descriptively. Differences between groups were performed using t-test or Mann-Whitney for continuous variables and Fisher’s exact test for categorical variables. All tests were performed at the 5% significance level. Statistical analysis was performed using SPSS 23.0 software.

## Results

### Patients’ characteristics and intraoperative details

There were no significant differences in baseline characteristics of both groups (Table 1). The overall success rate was 100% and no cases with positive surgical margins in both groups. The two groups did not show any statistically significant differences in terms of postoperative pathological grading, staging, and lymph node positivity rates. The MICUD group dissected a higher number of lymph nodes (23.3 ± 9.2 vs. 15 ± 6.4, *p* = 0.016). The total operative time was lower in the MICUD group (335.8 ± 39.3 mins vs. 413 ± 91.4 mins, *p* = 0.027). Postoperative pain scores at 4 and 24 h were lower in the MICUD group [3 (IQR: 2-3) vs. 5 (IQR: 4.75-6), *p* < 0.001 and 2 (IQR: 1-3) vs. 4 (IQR: 3-5), *p* < 0.001, respectively]. The MICUD group had shorter times for postoperative flatus [2 (IQR: 1-2) days vs. 3 (IQR: 3-3.25) days, *p* = 0.001], resumption of a semi-fluid diet [2 (IQR: 2-3) days vs. 3 (IQR: 3-4) days, *p* = 0.020], ambulation [1 (IQR: 1-2) day vs. 3 (IQR: 2-3) days, *p* = 0.004], and hospital stay [7 (IQR: 7-9) days vs. 12 (IQR: 7.75-25.25) days, *p* = 0.017]. The MICUD group had a lower VSS score [3 (IQR: 2.5-3) vs. 7 (IQR: 7-8.25), *p* < 0.001]. However, there were no statistical differences in cases requiring discharge with a drainage tube, time to remove the drainage tube, drainage volume 7 days after surgery (Table 2).

**Table 1.** Patients demographics.

Variable	MICUD (n = 21)	ECUD (n = 10)	*P* value
Age, years, mean (SD)	67.1 (6.9)	62.9 (12.6)	0.346
BMI, kg/m2, mean (SD)	25.2 (3.8)	25.1 (3.9)	0.944
aCCI, mean (SD)	5.4 (1.6)	4.6 (1.7)	0.192
ASA score, n (%)			> 0.9
1 ∼ 2	20 (95.2%)	9 (90%)	
3	1 (4.8%)	1 (10%)	
Diabetes, n (%)	6 (28.6%)	2 (20%)	> 0.9
Hypertension, n (%)	14 (66.7%)	5 (50%)	0.447
History of abdominal surgery, n (%)	7 (33.3%)	4 (40%)	> 0.9
Clinical stage, n (%)			0.458
<cT2	12 (57.1%)	4 (40%)	
≥cT2	9 (42.9%)	6 (60%)	
Neoadjuvant therapy, n (%)	10 (47.6%)	3 (30%)	0.452

BMI, body mass index; aCCI, age-adjusted Charlson comorbidity index; ASA, American Society of Anesthesiologists; MICUD, modified intracorporeal urinary diversion; ECUD, extracorporeal urinary diversion.

**Table 2.** Patients’ intraoperative details.

Variable	MICUD (*n* = 21)	ECUD (*n* = 10)	*P* value
Total operative time, mins, mean (SD)	335.8 (39.3)	413 (91.4)	0.027
Urethrectomy, n (%)	15 (71.4%)	4 (40%)	0.127
Preservation of reproductive organs, n (%)	3 (14.3%)	3 (30%)	0.358
Transfusion, n (%)	7 (33.3%)	2 (20%)	0.677
pT stage, n (%)			0.665
T0	4 (19.0%)	2 (20%)	
Ta ∼ T1	9 (42.9%)	4 (40%)	
T2	5 (23.8%)	1 (10%)	
T3	3 (14.3%)	2 (20%)	
T4	0 (0%)	1 (10%)	
Pathological grading, n (%)			> 0.9
Low	1 (4.8%)	0 (0%)	
High	16 (76.2%)	8 (80%)	
pN stage, n (%)			> 0.9
pN0	17 (81.0%)	8 (80%)	
pN+	4 (19.0%)	2 (20%)	
Total number of removed LNs, n, mean (SD)	23.3 (9.2)	15 (6.4)	0.016
Overall success rate (%)	100% (21/21)	100% (10/10)	
Pain scores, median (IQR)			
4h postoperatively	3 (2-3)	5 (4.75-6)	< 0.001
24 hours postoperatively	2 (1-3)	4 (3-5)	< 0.001
Time to flatus, d, median (IQR)	2 (1-2)	3 (3-3.25)	0.001
Time to intake of semi-fluid diet, d, median (IQR)	2 (2-3)	3 (3-4)	0.020
Time to ambulation, d, median (IQR)	1 (1-2)	3 (2-3)	0.004
Case requiring discharge with a drainage tube, n (%)	10 (47.6%)	3 (30%)	0.452
Time to remove the drainage tube, d, median (IQR)	13 (6.5-20.5)	13 (8.5-25.5)	0.497
Drainage volume 7 days after surgery, ml, median (IQR)	1655 (1093.5-2706.5)	2528 (1445.5-2926.25)	0.422
Post-operative hospital stays, d, median (IQR)	7 (7-9)	12 (7.75-25.25)	0.017
CCI, mean (SD)	23.5 (14.8)	29.5 (17.0)	0.317
VSS score, median (IQR)	3 (2.5-3)	7 (7-8.25)	< 0.001

LNs, lymph nodes; CCI, Comprehensive Complication Index; VSS, the Vancouver Scar Score; MICUD, modified intracorporeal urinary diversion; ECUD, extracorporeal urinary diversion.

### Learning curve

**Figure 2.**
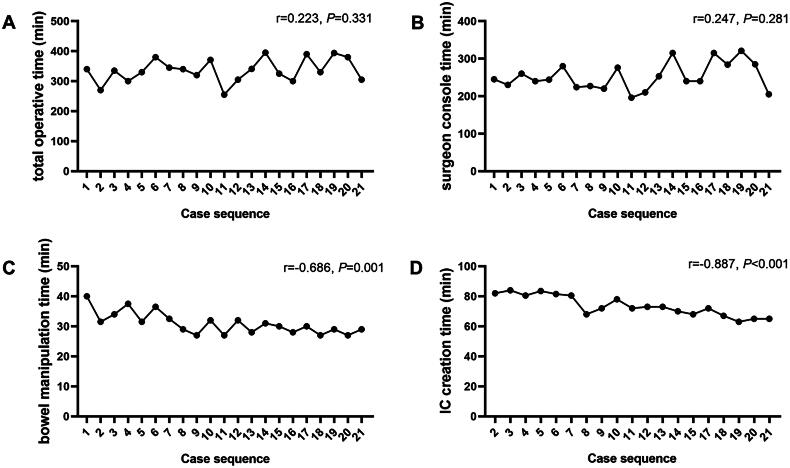
The learning curve of total operative time (A), surgeon console time (B), bowel manipulation time (C) and ileal conduit creation time (D). Since case 1 and case 16 have previously undergone radical nephroureterectomy, they are not plotted in the learning curve of ileal conduit (IC) creation time.

**Figure 3.**
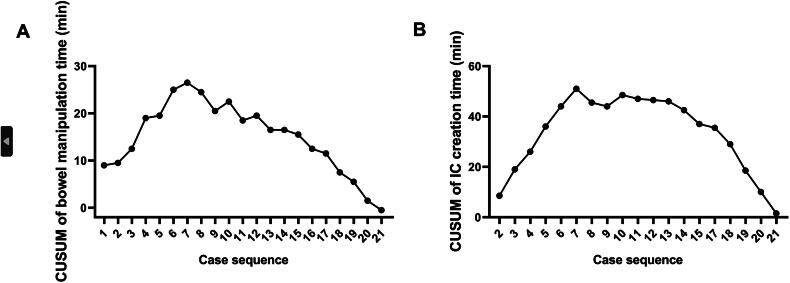
Learning curve based on cumulative summation (CUSUM) method of bowel manipulation time (A) and ileal conduit creation time (B). The inflection point was around the 7th case. Since case 1 and case 16 have previously undergone radical nephroureterectomy, they are not plotted in the learning curve of ileal conduit (IC) creation time.

For MICUD group, the total operation time and SCT do not show any significant correlation with the cumulative number of surgeries (*r* = 0.223, *p* = 0.331; *r* = 0.247, *p* = 0.281) (Figure 2A-B), which may be related to the surgeons having extensive experience with laparoscopic surgery. The time for bowel manipulation and IC construction decreases gradually with the increase in the number of operations (r =-0.887, *p* < 0.001; r=-0.686, *p* = 0.001) (Figure 2C-D). The CUSUM method was used to further plot the learning curve for bowel manipulation and IC construction. The phases of the learning curve, learning and proficiency, were then determined based on a steep drop-off at case number 7. The learning curve was divided into two distinct phases: the learning phase (cases 1-7) and the proficiency phase (cases 8-21) (Figure 3). During the learning phase, there was a noticeable increase in operative time and variability, reflecting the surgical team’s adaptation to the novel MICUD technique. As experience was gained, the proficiency phase demonstrated reduced operative times and greater consistency in performance. In this cohort, the respective times for total operative time, surgeon console time, radical cystectomy time (including pelvic lymph node dissection), TV-NOSES time, bowel manipulation time, and ileal conduit (IC) creation time were 335.8 ± 39.3 min, 252.9 ± 36.6 min, 167.3 ± 21.5 min, 16.1 ± 3.6 min, 31.0 ± 3.6 min, and 73.6 ± 6.8 min, respectively.

### Complications

The occurrence of postoperative complications within 30 days, whether classified according to the organ system involved or according to the Clavien-Dindo grading, did not show any significant difference between the two groups (Table 3). Additionally, the CCI was similar between the two groups as well (23.5 ± 14.8 VS. 29.5 ± 17.0, *p* = 0.317). In the MICUD group, 55 postoperative complications were observed in 18 (85.7%) patients, with 53 (96.4%) of these being minor complications and a median of 2 (IQR: 1-4) complications per patient. The most prevalent complications were vessels related (10/55, 18.2%). Conversely, in the ECUD group, 32 postoperative complications were observed in 9 (90%) patients, with 30 (93.8%) of these being minor complications and a median of 3 (IQR: 1-5.25) complications per patient. Infections were the most common type of complication (6/32, 18.8%) (Tables 3–5). In terms of vaginal-related complications, neither group experienced any short-term complications within 30 days postoperatively, nor any long-term complications within 30 to 90 days. One patient in the MICUD group developed an anterior vaginal wall hernia six months post-surgery.

**Table 3.** Complications according to the organ system involved and according to Clavien-Dindo classification within 30 d.

Complications within 30 d. No. (%)	All (31)	MICUD (21)	ECUD (10)	*P* value
Gastrointestinal	9 (29.0%)	5 (23.8%)	4 (40%)	0.417
Infectious	9 (29.0%)	5 (23.8%)	4 (40%)	0.417
Genitourinary	11 (35.5%)	6 (28.6%)	5 (50%)	0.423
Vessels	14 (45.2%)	10 (47.6%)	4 (40%)	> 0.9
Cardiac	2 (6.5%)	2 (9.5%)	0 (0%)	> 0.9
Hematopathy	11 (35.5%)	9 (42.9%)	2 (20%)	0.262
Any complication (grade 1-5)	27 (87.1%)	18 (85.7%)	9 (90%)	> 0.9
Minor complication (grade 1-2)	26 (83.9%)	17 (81.0%)	9 (90%)	> 0.9
Major complication (grade 3-5)	4 (12.9%)	2 (9.5%)	2 (20%)	0.577
≥grade 2 complication	23 (74.2%)	15 (71.4%)	8 (80%)	> 0.9
Total any-grade complications per patient, median (IQR])	2 (1-4)	2 (1-4)	3 (1-5.25)	0.335

MICUD, modified intracorporeal urinary diversion; ECUD, extracorporeal urinary diversion.

**Table 4.** Grading, therapeutic management, incidence and proportion of complications within 30 days of perioperative period in MICUD group.

	CDC grading	Management	Number of complications	Proportion, % (*n* = 21)
Gastrointestinal				
9 complications (16.4%) in 5 patients				
Incomplete intestinal obstruction	1	Conservative; cessation of oral intake and i.v. fluid support	1	4.8
Vomit	1	Conservative; antiemetics	3	14.3
Diarrhea	1	Conservative; antidiarrheals, i.v. fluid support, electrolytes	1	4.8
Abdominal distention	1	Conservative	3	14.3
Intestinal dysfunction	2	Probiotics; i.v. fluid support	1	4.8
Infectious				
5 complications (9.1%) in 5 patients				
Fever of unknown origin	2	Conservative; antipyretics, antibiotic treatment	1	4.8
Urinary tract infection	2	Foley catheter extraction; antibiotic treatment	1	4.8
Abdominal infection/peritonitis	2	antibiotic treatment	2	9.5
Pneumonia	2	antibiotic treatment	1	4.8
Genitourinary				
7 complications (12.7%) in 6 patients				
Urostomy ischemia	3b	Laparotomy and surgical revision	1	4.8
Hydronephrosis (new onset)	1	Conservative; clinical observation or diagnostic evaluation only	4	19.0
Acute kidney injury	1	Conservative; i.v. fluid support	2	9.5
Vessels				
10 complications (18.2%) in 10 patients				
Deep vein thrombosis	2	Anticoagulation	7	33.3
	3a	Placement of vascular filters	1	4.8
Lymphocele	1	Conservative; clinical observation or diagnostic evaluation only	2	9.5
Cardiac				
2 complications (3.6%) in 2 patients				
Arrhythmia	2	Conservative; medical cardioversion	1	4.8
Cardiac insufficiency	2	Medical treatment	1	4.8
Hematopathy				
9 complications (16.4%) in 9 patients				
Anemia requiring transfusion	2	Blood transfusion	7	33.3
Leucopenia	1	Conservative; clinical observation or diagnostic evaluation only	2	9.5
Miscellaneous				
13 complications (23.6%) in 8 patients				
Electrolyte disturbance	1	Conservative; electrolytes	4	19.0
Hypoproteinemia	2	Conservative; medical therapy	6	28.6
Acidosis	1	Conservative; medical therapy, electrolytes	1	4.8
Edema	1	Conservative; diuretics	1	4.8
Other rare complications	1	Conservative	1	4.8

MICUD, modified intracorporeal urinary diversion; CDC, Clavien-Dindo classification.

**Table 5.** Grading, therapeutic management, incidence and proportion of complications within 30 days of perioperative period in ECUD group.

	CDC grading	Management	Number of complications	Proportion, % (*n* = 10)
Gastrointestinal				
5 complications (15.6%) in 4 patients				
Vomit	1	Conservative; antiemetics	1	10
Intestinal dysfunction	2	Probiotics; i.v. fluid support	1	10
Abdominal distention	1	Conservative	2	20
Gastrointestinal bleeding	2	Medical treatment	1	10
Infectious				
6 complications (18.8%) in 4 patients				
Urinary tract infection	2	Foley catheter extraction; antibiotic treatment	3	30
Abdominal infection/peritonitis	2	antibiotic treatment	2	20
Pneumonia	2	antibiotic treatment	1	10
Genitourinary				
5 complications (15.6%) in 5 patients				
Urostomy ischemia	3b	Laparotomy and surgical revision	1	10
Hydronephrosis (new onset)	1	Conservative; clinical observation or diagnostic evaluation only	4	40
Vessels				
5 complications (15.6%) in 4 patients				
Deep vein thrombosis	2	Anticoagulation	2	20
Lymphocele	1 3a	Conservative; clinical observation or diagnostic evaluation only Drainage	2 1	20 10
Hematopathy				
2 complications (6.3%) in 2 patients				
Anemia requiring transfusion	2	Blood transfusion	2	20
Miscellaneous				
9 complications (28.1%) in 4 patients				
Electrolyte disturbance	1	Conservative; electrolytes	1	10
Hypoproteinemia	2	Conservative; medical therapy	4	40
Dizziness	1	Conservative; clinical observation or diagnostic evaluation only	1	10
Pain	1	Conservative; analgesics	3	30

ECUD, extracorporeal urinary diversion; CDC, Clavien-Dindo classification.

### Follow-up results and oncologic outcomes

The mean follow-up times were 29.3 and 22.6 months for the ECUD and MICUD groups, respectively. The reasons for readmissions in the MICUD group within 3 months postoperatively included one case of incomplete intestinal obstruction (Clavien-Dindo grade 1) and one case for retrieval of an inferior vena cava filter (Clavien-Dindo grade 3a). Additionally, one patient was readmitted within 6 months due to hypoproteinemia (Clavien-Dindo grade 2). In the ECUD group, two patients were readmitted within 3 months for urinary tract infections (Clavien-Dindo grade 2), and one patient was readmitted within 6 months for a pelvic abscess (Clavien-Dindo grade 3a). In the MICUD group, there was one case of local tumor recurrence, two cases of distant metastasis, including one death from progression; in the ECUD group, one patient had distant metastasis and died from tumor progression.

## Discussion

Urinary diversion, as an integral part of radical cystectomy, is a crucial step that affects patients’ postoperative recovery and the occurrence of complications. Surgeons tend to choose extracorporeal diversion, primarily due to concerns about increased complexity of bowel manipulation and extended surgical time with ICUD. Our standout feature and improvement lie in the utilization of the natural vaginal cavity as a conduit for Endo-GIA stapler, in contrast to the conventional ICUD approach, which typically involves establishing a conduit above the pubic symphysis or in the left lower abdomen [7,8]. By utilizing the vaginal route, the bowel descends naturally, leading to a significant enhancement in operational space and angles. This subsequently reduces the need for traction and clamping on the intestinal tube and its mesentery. Morizane et al. [18] reported an average bowel manipulation time of 46.7 min during intracorporeal IC construction. In our MICUD group, the corresponding time was 30.9 min, indicating that the modified technique may further reduce the impact time on the bowel. Previous study has suggested that bowel manipulation time may influence the incidence of postoperative ileus [19]. This approach simplifies the intestinal reconstruction process, making it easier to ensure precise blood supply and accurate anastomosis during the procedure. In fact, we found that the total operative time for MICUD group to be significantly shorter than ECUD group (335.8 ± 39.3 VS. 413 ± 91.4, *p* = 0.019). Patients in the MICUD group experienced a faster recovery of gastrointestinal function, with no gastric tube placement during the perioperative period. The median time for postoperative flatus and resumption of a semi-liquid diet was the second day after surgery. There were no occurrences of bowel fistulas, and only one case of incomplete bowel obstruction that improved with conservative treatment. Similarly, the MICUD can also be applied to the construction of intracorporeal neobladders.

The learning curve reflects how surgeons adapt to the new technique over time, and highlights the inflection point at which proficiency is achieved. In this study, the CUSUM analysis indicated that after the initial seven cases, the surgeon achieved a steady and efficient level of performance. Although our sample size was limited, the trends observed in the learning curve suggest that the MICUD technique can be efficiently learned and mastered within a relatively short period. We believe that this information is useful for surgeons considering the adoption of this technique, providing a preliminary reference for the number of cases needed to achieve proficiency. According to previous reports, the learning curve of ICUD is influenced by the patient-side surgeon [20]. In our cohort, the assistant was proficient in using the Endo-GIA stapler. Additionally, the MICUD technique provides the assistant with a larger working space and more ideal angles, making the operation easier and reducing the technical demands on the patient-side surgeon. This feature is also one of the key advantages of the MICUD approach.

Patients presently aspire to undergo procedures that not only treat diseases effectively but also minimize trauma and promote aesthetic wound appearance. Due to the excellent extensibility and reparative capability of the vaginal wall, TV-NOSES has become feasible [21,22]. This approach avoids the need for a large abdominal incision and associated complications. Meanwhile, it significantly reduces postoperative pain and shortens the recovery time [22,23]. Moreover, with the modified intracorporeal approach, the surgical trauma may be further reduced. In the MICUD group, the patients’ postoperative pain scores and time to ambulation were significantly better than those in the ECUD group. The postoperative Vancouver Scar Scale was 3 (IQR: 2.5-3) points, indicating excellent healing of the conduit incision, which closely resembled the surrounding normal skin tissue. For sexually active patients who wish to preserve their reproductive organs, a transvaginal approach through the posterior cul-de-sac can be employed to retrieve pathological specimens from the posterior vaginal wall, safeguarding the nerves concentrated in the anterior vaginal wall [24]. In our study, most patients had both their ovaries and uterus removed along with the bladder. One patient chose to preserve her uterus, and two preserved their ovaries in the MICUD cohort. The patient who preserved her uterus did not experience any vaginal complications, tumor remnants, or recurrence during follow-up. It is worth noting that another patient in the MICUD group experienced adipose tissue prolapse in the anterior vaginal wall six months after the surgery. The patient subsequently underwent pelvic mass resection and vaginal wall repair surgery. We consider that this complication might be related to factors such as the patient’s obesity (BMI: 29.9), long-term constipation, and engaging in physically demanding activities. To reduce the occurrence of similar incidents, we emphasize the protection of the anterior vaginal wall during surgery. When the anterior vaginal wall is resected, we can use the clamshell closure technique with a running suture for vaginal reconstruction. If clamshell closure proves to be challenging during vaginal reconstruction, the Mercedes-Benz closure serves as an effective alternative approach [15]. Apart from this particular case, no other patients experienced vaginal-related complications.

Regarding postoperative complications, the lack of a unified standard definition for specific complications leads to significant variations among evaluators, resulting in widely different research outcomes. For instance, Wijburg et al. [25] reported a 49% incidence rate of postoperative complications within 30 days of undergoing RARC. Vetterlein et al. [26] conducted a study on 506 patients who underwent open radical cystectomy, finding that 99% of the patients experienced complications of varying degrees, suggesting a serious underestimation of complications in previous reports. Mendrek et al. [27] indicated a 92% incidence rate of postoperative complications in patients undergoing RARC. However, a higher proportion of complications does not necessarily indicate poorer medical standards. Instead, detailed recording and standardized examinations enable more effective comparisons between different researches. Therefore, we utilize the Clavien-Dindo classification and adhere to the EAU guidelines panel assessment and recommendations [28]. In MICUD group, a total of 55 complications were observed in 18 patients within 30 days after surgery, but 53 (96.4%) of them were minor complications. Our research also indicated that MICUD did not increase the incidence of wound or pelvic infections caused by intestinal bacterial dissemination. There were no significant differences in the incidence of postoperative complications between the two groups whether classified according to the organ system involved or according to the Clavien-Dindo grading. The postoperative conditions of this group further confirmed the safety of our MICUD approach, indicating that MICUD is as safe and feasible as traditional ECUD. Additionally, patients in the MICUD group had faster postoperative recovery, shorter hospital stay.

This study has some limitations. Firstly, the sample size is relatively small. Secondly, it is a retrospective cohort study rather than a randomized trial, hence there is potential for information bias. Despite these limitations, we believe that our modified technology demonstrates a safe and feasible option for intracorporeal ileal conduit construction.

## Conclusions

The MICUD technique for constructing the IC is simple, with ideal operative space and angles, making it easy to learn. Compared to ECUD, patients experience less trauma, faster recovery and shorter length of hospital stay. In terms of postoperative complications and oncological efficacy, both methods are similar. Therefore, RARC with MICUD represents a safe and feasible minimally invasive surgical approach.

## Supplementary Material

Supplemental Material

## Data Availability

The raw data of this study and more information can be found upon reasonable request from the corresponding author HH (email: huhailong@tmu.edu.cn).
