# Supplementary material for: Utilizing vaginal natural orifice to facilitate bowel manipulation during totally intracorporeal ileal conduit construction: a retrospective cohort study
Source: Ann Med. 2025 Jan 18;57(1):2453827. doi: 10.1080/07853890.2025.2453827 (PMC11748861; doi:10.1080/07853890.2025.2453827)

**Utilizing Vaginal Natural Orifice to Facilitate Bowel Manipulation During Totally Intracorporeal Ileal Conduit Construction: A Retrospective Cohort Study**

**Kaipeng Jia^1,2^, Shiwang Huang^1,2^, Zhun Wang^1,2^, Yuda Lin^1,2^, Yiduo Bai^1,2^, Chong Shen^1,2^, Zhe Zhang^1,2^, Zhouliang Wu^1,2^, Yunkai Qie^1,2^*, Hailong Hu^1,2^***

^1^Department of Urology, The Second Hospital of Tianjin Medical University, Tianjin, China
^2^Tianjin Key Laboratory of Urology, Tianjin Institute of Urology, The Second Hospital of Tianjin Medical University, Tianjin, China

Kaipeng Jia, Shiwang Huang Contribute equally to this work as co-first authors.

***Co-corresponding authors:**

Yunkai Qie
Department of Urology, The Second Hospital of Tianjin Medical University, 23 Pingjiang Road, Hexi District, Tianjin, 300211, People's Republic of China.
E‑mail: [qieyunkai@tmu.edu.cn](mailto:qieyunkai@tmu.edu.cn)

Hailong Hu
Department of Urology, The Second Hospital of Tianjin Medical University, 23 Pingjiang Road, Hexi District, Tianjin, 300211, People's Republic of China.
E‑mail: [huhailong@tmu.edu.cn](mailto:huhailong@tmu.edu.cn)

**The port placements were as follows:**

Following the establishment of pneumoperitoneum, an 8 mm trocar was inserted at a distance of 2 cm above the umbilicus as the camera arm port for the second robotic arm. On both sides, along the anterior axillary line and parallel to the observed camera ports, 8 mm trocars were respectively placed. These trocars were used to connect the first and fourth robotic arms. At approximately 8 cm away from the ports of the second and fourth robotic arms, trocars were inserted at the predetermined stoma site for the connection of the third robotic arm. Below the left costal margin, at a distance of approximately 8 cm from the ports of the first and second robotic arms, a 12mm trocar was placed as the port for the assistant (**Supplementary Figure 1**).

**Figure legends**

**Supplementary Figure 1** The patient’s position and trocar placement. **A** The patient was placed in the Trendelenburg lithotomy position. **B** The trocar placement: b trocar was established as the camera arm port for the second robotic arm; a, c, and d trocars were used to connect the first, third, and fourth robotic arms, respectively; e trocar was established as the port for the first assistant.


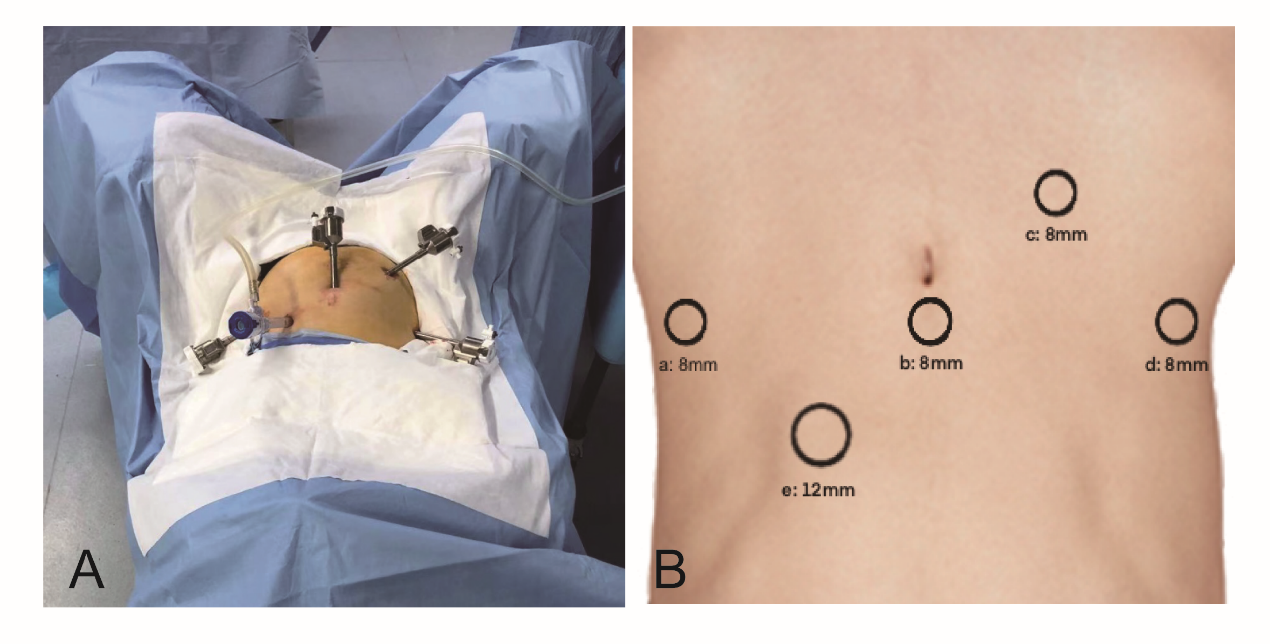

Supplement: Supplemental Material [file IANN_A_2453827_SM8585.docx]
